# Investigating the “Dark” Genome: First Report of Partington Syndrome in Cyprus

**DOI:** 10.3390/genes16101224

**Published:** 2025-10-15

**Authors:** Constantia Aristidou, Athina Theodosiou, Pavlos Antoniou, Angelos Alexandrou, Ioannis Papaevripidou, Ludmila Kousoulidou, Pantelitsa Koutsou, Anthi Georghiou, Türem Delikurt, Elena Spanou, Nicole Salameh, Paola Evangelidou, Kyproula Christodoulou, Alain Verloes, Violetta Christophidou-Anastasiadou, George A. Tanteles, Carolina Sismani

**Affiliations:** 1 Department of Clinical Genetics and Genomics, The Cyprus Institute of Neurology and Genetics, 2371 Nicosia, Cyprus; 2 Department of Cytogenetics and Genomics, The Cyprus Institute of Neurology and Genetics, 2371 Nicosia, Cyprus; 3 Waltham Petcare Science Institute, Melton Mowbray, Leicestershire LE14 4RT, UK; 4 Neurogenetics Department, The Cyprus Institute of Neurology and Genetics, 2371 Nicosia, Cyprus; 5 Genetics Department, APHP-Robert Debré University Hospital, Université Paris Cité, 75019 Paris, France; 6 Genetics Clinic, Karaiskakio Foundation, 2032 Nicosia, Cyprus; 7 Department of Basic and Clinical Sciences, University of Nicosia Medical School, 2408 Nicosia, Cyprus

**Keywords:** ARX, ARXdup24, Partington syndrome, X-linked intellectual disability, dark genomic regions

## Abstract

**Background/Objectives**: X-linked intellectual disability (XLID) is a highly heterogeneous disorder accounting for ~10% of all males with ID. Next-generation sequencing (NGS) has revolutionized the discovery of causal XLID genes and variants; however, many cases remain unresolved. We present a four-generation syndromic XLID family with multiple males exhibiting variable degree of ID, focal dystonia and epilepsy. **Methods**: Extensive cytogenetic and targeted genetic testing was initially performed, followed by whole-exome sequencing (WES) and short-read whole-genome sequencing (WGS). Apart from the routine NGS analysis pipelines, sequencing data was revisited by focusing on poorly covered/mapped regions on chromosome X (chrX), to potentially reveal unidentified clinically relevant variants. Candidate variant validation and family segregation analysis were performed with Sanger sequencing. **Results**: All initial diagnostic testing was negative. Subsequently, 300 previously reported “dark” chrX coding DNA sequences, overlapping 97 genes, were cross-checked against 29 chrX genes highly associated (*p* < 0.05) with ID and focal dystonia, according to Phenomizer. Manual inspection of the existing NGS data in two low-coverage regions, chrX:25013469-25013696 and chrX:111744737-111744820 (hg38), revealed a recurrent pathogenic *ARX* variant NM_139058.3:c.441_464dup p.(Ala148_Ala155dup) (ARXdup24) associated with non-syndromic or syndromic XLID, including Partington syndrome. Sanger sequencing confirmed ARXdup24 in all affected males, with carrier status in their unaffected mothers, and absence in other unaffected relatives. **Conclusions**: After several years of diagnostic odyssey, the pathogenic ARXdup24 variant was unmasked, supporting a genotype–phenotype correlation in the first Partington syndrome family in Cyprus. This study highlights that re-examining underrepresented genomic regions and using phenotype-driven tools can provide critical diagnostic insights in unresolved XLID cases.

## 1. Introduction

Intellectual disability (ID) is a genetically heterogeneous neurodevelopmental disorder characterized by significant limitations in both cognitive functioning and adaptive behaviour. X-linked intellectual disability (XLID) is caused by mutations in genes located on chromosome X (chrX) and accounts for ~10% of ID in males [1,2]. Based on the clinical presentation, XLID can be categorised as non-syndromic when isolated, or as syndromic when it presents with one or multiple clinical features or co-morbidities. Fragile X syndrome (FXS), typically caused by an expansion of the CGG triplet repeat (>200) in the fragile X messenger ribonucleoprotein 1 (*FMR1*) gene on Xq27.3, is the most common inherited form of intellectual disability, and the leading form of XLID [2]. Apart from FXS, around 200 XLID syndromes have been characterized to date, while thus far, 172 XLID genes have been identified, accounting for 157 of these syndromes [3,4].

The conventional strategies for the identification of XLID and/or associated genes/conditions include family pedigree analysis [5], linkage analysis [6], chromosomal analysis often coupled with fluorescence in situ hybridization (FISH) [7], as well as array-based methods [8,9,10]. Especially in carriers of balanced X-autosome translocations, fine-mapping of the breakpoints has been instrumental for the characterization of candidate genes implicated in XLID [11]. Next-generation sequencing (NGS) revolutionized the discovery of XLID genes and gene variants by assessing the chrX, exome or genome of patients in an efficient, cost-effective and high-throughput manner [3,4,12,13,14,15,16]. Despite the finer resolution and deeper mechanistic insight offered by the evolving XLID research methods over time, many patients with syndromic or non-syndromic XLID remain without a genetic diagnosis. This diagnostic pitfall could be due to the involvement of yet to be discovered novel genes, or the presence of non-coding or regulatory causative variants, such as deep intronic splice variants, that cannot be captured by whole-exome sequencing (WES) or are not confidently supported even if identified by whole-genome sequencing (WGS) [17]. The presence of single-nucleotide variants (SNVs), structural variants or complex rearrangements often missed by short-read NGS methods, due to coverage and mapping limitations, could also be another explanation [17,18,19].

In 2019, Ebbert et al. demonstrated the existence of approximately 37 thousand “dark” regions throughout the genome using short-read NGS technologies; that is, genomic regions with few or no mappable reads (dark-by-depth) or genomic regions with aligned reads but with low mapping quality (dark-by-mapping quality (MAPQ)) [20]. Such “dark” genomic regions span protein-coding exons from ~800 genes involved in many biological pathways relevant to human health and development, while ~10% of these genes are associated with known human diseases [20]. For example, the Survival of motor neuron 1 (*SMN1*) and Survival of motor neuron 2 (*SMN2*) genes (“dark” by 94% and 88%, respectively), both associated with spinal muscular atrophy (OMIM * 600354 and * 601627), the Aristaless-related homeobox (*ARX*) gene (“dark” by 12.8%) associated with a spectrum of X-linked developmental disorders including Partington syndrome (OMIM * 300382), and the T-box transcription factor 1 (*TBX1*) gene (“dark” by 10.6%) associated with the 22q11.2 deletion syndrome (OMIM * 602054), are all located within “dark” genomic regions [20]. Since disease-causing variants in such regions often escape the detection capabilities of standard short-read NGS methods, focused re-evaluation of existing NGS data in unresolved cases could be useful before proceeding with alternative diagnostic approaches.

We present a four-generation family with a suspected syndromic XLID disorder that remained undiagnosed despite extensive cytogenetic and molecular testing, including WES and short-read WGS. This study aims to perform an NGS data reanalysis, focusing on “dark” genomic regions on chrX, to potentially unmask any hidden variants in underrepresented genomic regions implicated in XLID.

## 2. Materials and Methods

### 2.1. Family Description and Clinical Evaluation

**Figure 1:**
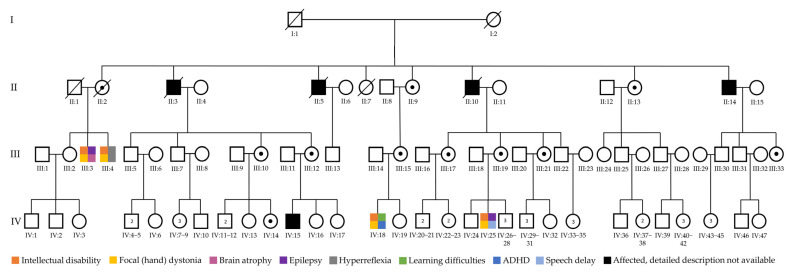
Schematic pedigree representation of the four-generation family with a suspected X-linked disorder. Affected males are indicated with colour-filled squares, while non-affected carrier females are marked with a dot inside a circle.

The family presented here included nine affected males across four generations; however, a thorough clinical evaluation was initially performed in four of the affected males (III:3, III:4, IV:18 and IV:25) (Figure 1). Three male family members were deceased (II:3, II:5 and II:10), while a detailed clinical description was not available for the remaining affected individuals (II:14 and IV:15). All four clinically evaluated patients had variable ID, epilepsy and movement abnormalities that included focal (hand) dystonia (Figure 1). The high incidence of affected male individuals in the family, the absence of phenotype in the mothers of affected males, and the absence of male-to-male transmission suggested an underlying X-linked disorder.

### 2.2. Cytogenetic and Initial Molecular Testing

Chromosomal analysis was performed from heparinized peripheral blood samples using the conventional G-banding technique at an average resolution of 550 bands. Peripheral blood in EDTA tubes was subjected to DNA extraction using the QIAamp DNA Blood Midi kit (Qiagen, Hilden, Germany) according to the manufacturer’s protocol.

In order to investigate the possibility of small copy number changes (deletions/duplications) that are beyond the resolution of chromosomal analysis, array-CGH was also performed with two types of arrays. An upgraded locally-designed chrX exon specific array was used first, with over 90,000 oligonucleotide probes on chrX (Oxford Gene Technology, Kidlington, UK) [9], followed by a genome-wide CytoChip Oligo Array with 105,000 oligonucleotides (version 1.0—BlueGnome, Cambridge, UK). Array images were acquired using the Agilent microarray scanner G2565B (Agilent, Santa Clara, CA, USA), while fluorescent ratios were calculated using either the CytoSure Interpret Software (Oxford Gene Technology) or the BlueFuse Multi software (version 4.2—BlueGnome).

Fifty nanograms of the extracted DNA was used for Fragile X testing. Briefly, the number of the tandem (CGG) repeats at the *FMR1* gene was determined using the appropriate forward (5′-GCTCAGCTCCGTTTCGGTTTCACTTCCGGT-3′—FAM labeled) and reverse (5′-AGCCCCGCACTTCCACCACCAGCTCCTCCA-3′) primers and the HotStarTaq Plus DNA Polymerase (Qiagen), following the manufacturer’s recommendations. The fluorescently labelled amplicon (281bp) was then mixed with Hi-Di™ Formamide (Applied Biosystems, Thermo Fisher Scientific, Waltham, MA, USA), and the GeneScan™ 500 LIZ™ dye Size Standard (Applied Biosystems), followed by brief denaturation and fragment analysis on the 3130xl Genetic Analyzer (Applied Biosystems).

### 2.3. Next-Generation Sequencing

WES was performed on samples from individuals III:3 and III:4 (affected siblings), as a service by Oxford Gene Technology, using the SureSelect DNA—Human All Exon Kit (version 1.2) (Agilent), according to the manufacturer’s recommendations. The enriched libraries for each sample were quantified using the qPCR NGS Library Quantification Kit (Agilent), followed by pooling and 100bp paired-end sequencing (TruSeq v3 mode) on the HiSeq2000 platform (Illumina Inc., San Diego, CA, USA). WES data was analysed using an in-house bioinformatics exome analysis pipeline as described previously [21,22]. WES data analysis was initially focused on high/medium impact variants (i.e., splice site, frameshift, nonsense, stop-loss, missense) (>20× coverage) overlapping XLID genes, as found in the OMIM^®^ database (https://www.omim.org/), GeneCards database (https://www.genecards.org/), and XLID gene list by the Greenwood Genetic Centre (https://ggc.org/). A CNV analysis using the WES datasets from the affected siblings was performed using CoNIFER (version 0.2.2) [23].

Short-read WGS was performed on samples from individuals III:3, III:4, II:1 and II:2, as a service by Macrogen Europe, using the TruSeq DNA PCR-free kit (Illumina), according to the manufacturer’s recommendations. Paired-end sequences (2 × 150 bp) generated by the NovaSeq6000 platform (Illumina) were aligned to the human genome assembly GRCh38 (hg38) using the Isaac aligner (version 04.18.11.09) [24]. SNVs and short insertions and deletions (indels), structural variants and large indels, and CNVs were identified using Strelka (version 2.9.10) [25], Manta (version 1.5.0) [26], and Control-FREEC (version 11.5) [27], respectively. Variant annotation and effect prediction were performed using SnpEff (version 4.3t) [28] and an in-house program by Macrogen Europe. WGS data analysis was initially focused on chrX variants commonly found between the affected male siblings tested and inherited in an X-linked manner.

### 2.4. Reanalysis of “Dark” Genomic Regions

In order to potentially reveal unidentified clinically relevant variants in “dark” genomic regions, the existing NGS data was reanalysed focusing on poorly covered (dark-by-depth) or poorly mapped (dark-by-MAPQ) regions previously published by Ebbert et al., 2019 [20]. Specifically, one of the authors’ publicly available datasets was downloaded (additional file 3 in ref. [20]), which includes “dark” genomic regions either by depth or by mapping quality based on whole-genome Illumina sequencing data (100bp read lengths). These were initially filtered to retain only “dark” coding DNA sequences (CDS) on chrX (File S1).

The Phenomizer web-based application (https://hpo.jax.org/tools/phenomizer) of the Human Phenotype Ontology (HPO) database (https://hpo.jax.org/) was also used in order to identify candidate diseases and implicated genes that best explain common clinical features present in multiple affected members of the family [29,30]. Briefly, the HPO terms intellectual disability (HP:0001249) and focal dystonia (HP:0004373) were entered in Phenomizer and an X-linked inheritance (HP:0001417) was selected, as suspected from the family pedigree. Only candidate diseases and implicated genes highly associated (*p* < 0.05) with the submitted HPO terms were taken into consideration for our focused short-read NGS data reanalysis (highlighted in red in File S1).

The resulting “dark” chrX CDS were then cross-checked with the list of genes highly associated with intellectual disability and focal dystonia. To do so, simple formulas were used to retain single values in each of the two datasets, followed by a comparison of the resulting data to identify common genes between the two (columns C and E in “Dark x Phenomizer” tab of File S1). That is, genes on chrX highly associated with intellectual disability and focal dystonia that fully or partially overlap “dark” CDS. The resulting targeted regions were manually inspected in the existing NGS data of the patients, using the Integrative Genomics Viewer (IGV), to reveal clinically relevant variants that may have remained undetected.

### 2.5. Sanger Sequencing Validation and Family Segregation Analysis

In order to validate the NGS reanalysis findings, PCR primer pairs (Metabion, Planegg, Germany) were designed flanking the region of interest using the Primer3 web interface tool (version 4.1.0) (https://primer3.ut.ee/) [31]. PCR amplification was then performed using the appropriate forward (5′-GTTCGAGGCCGAGCTGCAC-3′) and reverse (5′-CTCCTCCGGGTGCGTGAC-3′) primers, as well as the HotStarTaq Plus DNA Polymerase (Qiagen), following the manufacturer’s recommendations. Amplified products were visualized on a 2% agarose gel, which was stained with 1× GelRed^®^ Nucleic Acid Gel Stain (Biotium, Fremont, CA, USA). PCR products with the expected fragment size were purified using ExoSAP-IT^®^ (Affymetrix, Santa Clara, CA, USA), followed by cycle sequencing using the BigDye™ Terminator v1.1 Cycle Sequencing Kit (Applied Biosystems) and following the manufacturer’s recommendations. Cycle sequencing reaction clean-up was performed using Performa^®^ DTR Gel Filtration Cartridges (EdgeBio, Gaithersburg, MD, USA), and purified sequencing reactions were processed on the 3130xl Genetic Analyzer (Applied Biosystems). Sanger sequencing electropherograms were visually inspected using the ABI Sequencing Analysis Software (version 5.4), while resulting sequences were aligned to the human genome assembly GRCh38 (hg38) using the BLAT tool from the UCSC Genome Browser (https://genome.ucsc.edu/cgi-bin/hgBlat) [32].

### 2.6. Gene and Variant Nomenclature

Genes are described according to the HUGO Gene Nomenclature Committee (HGNC) guidelines [33]. All candidate variants are described according to the latest Human Genome Variation Society (HGVS) recommendations (https://hgvs-nomenclature.org/stable/) [34]. Finally, unless specified otherwise, all genomic coordinates in the present manuscript are based on the human genome assembly GRCh38 (hg38).

## 3. Results

### 3.1. Clinical Findings and Initial Testing Results

The family reported here includes nine affected males with a suspected X-linked disorder (Figure 1). The presented results are focused on the affected siblings III:3 and III:4, since they have gone through detailed clinical evaluation and comprehensive cytogenetic/molecular investigation in this study (Table 1).

Chromosomal G banding analysis in the two siblings (III:3 and III:4) revealed an apparently normal male karyotype. Subsequent array-CGH did not reveal any copy number changes other than CNVs in known polymorphic regions or CNVs inherited from a healthy parent, suggesting an apparently normal constitution. Genomic DNA analysis using PCR, determined no expansion of the tandem CGG repeats at the *FMR1* gene; these were within normal limits (<55 CGG) in both male siblings (Table 1). Finally, both WES and WGS analyses were negative; no candidate SNVs, CNVs, or SVs were detected, by the standard analysis pipelines, that could follow the expected inheritance pattern and/or explain the patients’ phenotypes.

### 3.2. Unmasking Clinically Relevant “Dark” Genomic Regions

Since the family remained undiagnosed despite extensive cytogenetic and molecular testing, the NGS data was reanalysed focusing on poorly covered (dark-by-depth) and poorly mapped (dark-by-MAPQ) regions on chromosome X, given the suspected X-linked disorder. The publicly available dataset by Ebbert et al., 2019, includes in total 36,794 “dark” genomic regions based on whole-genome Illumina sequencing data (100bp read lengths) [20]. Filtering for coding DNA sequences, present only on chrX, reduced this number to 300 “dark” regions, partially overlapping 97 genes.

**Figure 2:**
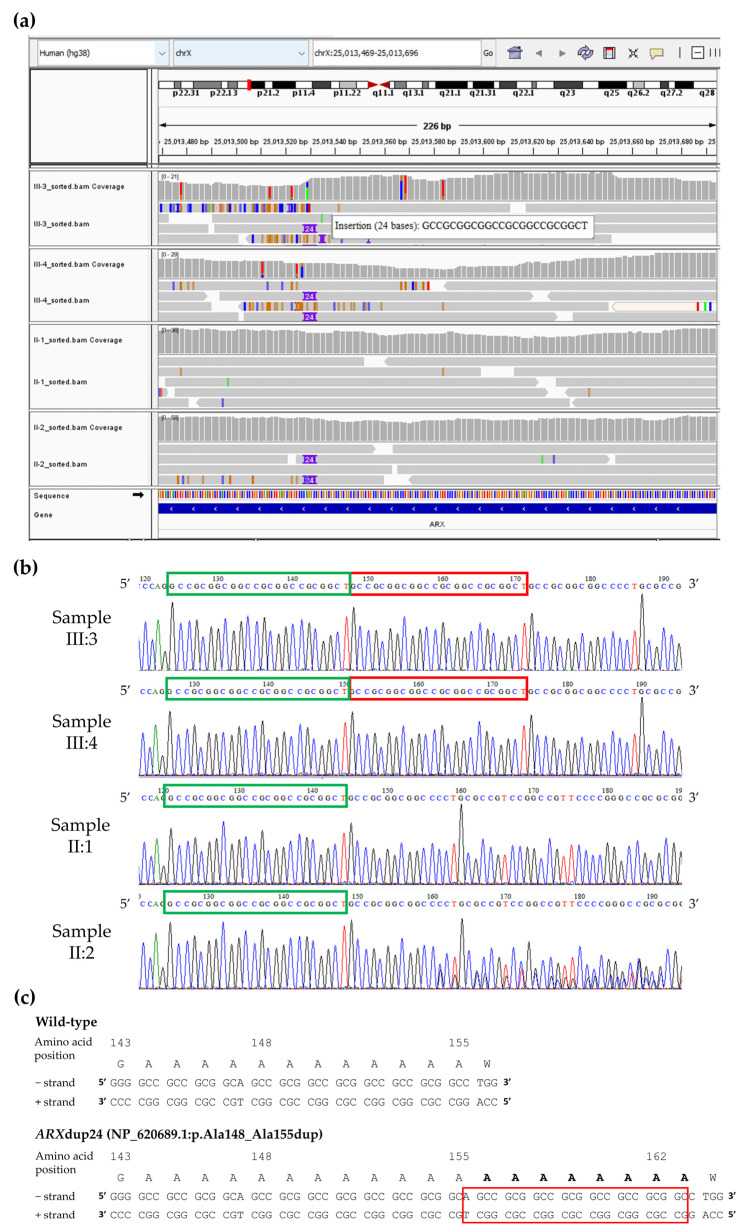
(**a**) IGV screenshot of the dark-by-depth region chrX:25013469-25013696 indicating the presence of a 24bp duplication (highlighted as an insertion in IGV) overlapping the *ARX* gene (III:3 = affected male; III:4 = affected sibling; II:1 = non-affected father; II:2 = non-affected mother). (**b**) Partial electropherograms confirming the ARXdup24 variant (red box) in the affected siblings (III:3 and III:4). The non-affected mother (II:2) is a carrier of ARXdup24, whereas ARXdup24 was not detected in the non-affected father (II:1). The electropherogram sequences displayed are from the reverse PCR reaction (plus strand) in a 5′→3′ orientation. (**c**) The NP_620689.1:p.(Ala148_Ala155dup) variant (red box) is predicted to result in an expansion of the ARX polyalanine track by adding eight alanine amino acids (bold letters).

A crosscheck performed between these genes and the Phenomizer list of 29 X-linked genes highly associated (*p* < 0.05) with intellectual disability (HP:0001249) and focal dystonia (HP:0004373), resulted in two “dark” regions (highlighted in green in File S1). The first is chrX:25013469-25013696 (hg38) overlapping exon 2 of the Aristaless-related homeobox (*ARX*) gene (OMIM *300382) (NM_139058.3), while the second is chrX:111744737-111744820 (hg38) overlapping exon 24 of the ALG13 UDP-N-acetylglucosaminyltransferase subunit (*ALG13*) gene (OMIM *300776) (NM_001099922.3). Manual inspection of the resulting regions in the existing short-read WGS data from the affected male siblings III:3 and III:4, and their non-affected parents II:1 and II:2, did not reveal any candidate variants disrupting *ALG13*; however, a possible 24bp duplication overlapping *ARX* (referred here as ARXdup24) was selected for further investigation (Figure 2a). Remarkably, there was no indication of the ARXdup24 variant when retrospectively reviewing the WES data of the affected siblings (III:3 and III:4) at the corresponding genomic region in hg19 (chrX:25031586-25031813).

PCR using primers flanking ARXdup24 confirmed the identified variant in a hemizygous state in the affected male siblings (III:3 and III:4) (Figure 2b). The non-affected mother (II:2) was a carrier of ARXdup24, whereas ARXdup24 was not detected in the non-affected father (II:1) (Figure 2b). Additional family members were subsequently tested, demonstrating presence of the ARXdup24 variant in three other affected males (IV:15, IV:18, IV:25), heterozygosity in their non-affected mothers (III:12, III:15, III:19) and a number of non-affected female relatives (II:9, II:13, III:10, IV:14), and absence in the remaining non-affected male or female individuals tested (III:14, III:24, IV:1, IV:2, IV:3).

The ARXdup24 variant, reported here as NM_139058.3:c.441_464dup according to the HGVS nomenclature recommendations [34], is predicted to result in an expansion of the ARX polyalanine track adding eight more alanine amino acids in the ARX protein NP_620689.1:p.(Ala148_Ala155dup) (Figure 2c).

## 4. Discussion

XLID is a highly heterogeneous neurodevelopmental disorder caused by direct or indirect disruptions of genes on chromosome X. Despite various technological advancements for detecting such causal chrX variants, many patients remain undiagnosed after routine genetic testing, even when short-read high-throughput sequencing methods are employed [13,14,15,16]. One reason for this may be the presence of “dark” regions in the genome, which cannot be successfully assembled or aligned using short-read NGS approaches, thus hindering the detection of clinically relevant gene variants within these underrepresented regions [20,35]. In the present study, a short-read NGS data reanalysis was performed in an undiagnosed XLID family focusing on such “dark” genomic regions. This enabled the unmasking of NM_139058.3:c.441_464dup p.(Ala148_Ala155dup) (ARXdup24), a recurrent 24bp in-frame duplication overlapping exon 2 of the *ARX* gene (NM_139058.3).

*ARX*, mapping on chromosome Xp21.3, is composed of 5 protein coding exons and is predominantly expressed in fetal brain, as well as adult skeletal muscle, heart, pancreas and liver [36,37]. It encodes the 562-amino acid Aristaless-related homeobox protein (NP_620689.1), which is composed of an N-terminal octapeptide domain, three nuclear localization sequences, four polyalanine tracts, an acidic domain, a homeobox domain, and a C-terminal aristaless domain [36]. ARX acts as a bifunctional transcriptional repressor and activator involved in the development of the brain as well as differentiation and maintenance of neurons in the cerebral cortex [36,38,39,40].

*ARX* is one of the most frequently mutated XLID genes, with at least 44 different variants reported to date (summarised in [41,42,43]). These range from point mutations (splice, nonsense, and missense variants) to indels and duplications, and cluster mainly within exon 2 of *ARX*. ARXdup24 specifically maps in a mutation hot spot in the second ARX polyalanine tract and is the most common variant, representing >40% of all reported *ARX* variants, followed by NM_139058.3: c.315_335dup p.(Ala109_Ala115dup), a similar duplication variant mapping in the first ARX polyalanine tract [41,43]. Despite its high frequency, the detection of ARXdup24 can be easily missed using a non-targeted approach such as short-read high-throughput sequencing. This diagnostic pitfall could be due to *ARX* having an unusually high GC content (~73%) and 12.8% “dark” CDS [20], particularly overlapping the first part of exon 2 where the recurrent ARXdup24 variant maps. The use of long-read sequencing technologies can substantially reduce the number of “dark” genomic regions [20,44], including those overlapping the *ARX* gene. However, validation by additional studies is required to support this further.

The identified ARXdup24 variant is classified here as pathogenic based on the Association for Clinical Genomic Science (ACGS) guidelines for variant classification in rare disease 2024 [45]. The criteria PS3_strong, PS4_strong, PM2_moderate and PP1_supporting were used to support this classification (ClinVar accession number: SCV006327456). The impact of ARXdup24 has been demonstrated in various functional studies (PS3_strong) supporting a partial loss of function of ARX. Specifically, a less striking reduction was observed in the transactivation and binding of ARX to the regulatory region of lysine-specific demethylase 5C (KDM5C) (OMIM * 314690), an XLID protein directly regulated by ARX and involved in chromatin remodeling and transcriptional repression [46]. Altered repression activity of the mutant ARX protein affecting the expression of transcriptional targets of ARX has also been demonstrated in an in vitro study after introducing the ARXdup24 variant [40]. Furthermore, the prevalence of ARXdup24 in affected individuals is significantly increased compared to the prevalence in controls (PS4_strong), as it has been identified in several affected individuals reported both in ClinVar (VCV000096455.23) and in the literature. It also has an extremely low frequency in controls (0.000002457) according to the Genome Aggregation Database (gnomAD v4.1.0) (PM2_moderate). Finally, it has been shown that ARXdup24 segregates with disease in related individuals, as also demonstrated in this study (PP1_supporting).

*ARX* variants, including ARXdup24, are associated with remarkable pleiotropy and a diverse spectrum of neurological phenotypes [47,48]. The majority of patients with ARXdup24 display non-syndromic ID (OMIM # 300419) [49], while the remaining present with West syndrome (OMIM # 308350) characterised by developmental and epileptic encephalopathy [50], and Partington syndrome (OMIM # 309510) characterised by syndromic XLID in conjunction with dystonic movement of the hands [51,52]. Partington syndrome is an X-linked neurodevelopmental disorder first described in a large Australian family by Partington et al., 1988 [51]. All affected males had mild to moderate ID and hand dystonia, inherited in an X-linked recessive pattern, while other phenotypes such as dysarthria, abnormal gait, autistic features and epilepsy were variable between individuals. The disease locus was initially mapped to the Xp21-Xpter chromosomal region, but this was later refined to Xp22.1 [51,53]. Fine mapping of the Partington syndrome locus, as well as identification of the causal *ARX* gene and ARXdup24 variant was accomplished in 2002 [36,38].

The patients in the family reported here (III:3, III:4, IV:18 and IV:25), in which ARXdup24 was found in a hemizygous state, commonly presented with mild ID and the characteristic dystonic movement of the hands, all of which are typical of Partington syndrome [47,52,54,55,56]. In addition, similarly to previous published cases, variable intra-familial phenotypic expressivity was also observed; for example, only two patients (III:3 and IV:25) were reported to have epilepsy. Finally, all female ARXdup24 carriers tested in the present study (II:2, II:9, II:13, III:10, III:12, III:15, III:19, and IV:14) were asymptomatic. This is in concordance with previous reports supporting the rarity of clinically affected ARXdup24 female carriers, whereas females carrying either truncating or missense *ARX* variants have been described presenting with a variable clinical spectrum (reviewed in [57]).

## 5. Conclusions

After several years of diagnostic odyssey, the pathogenic ARXdup24 variant was unmasked in an XLID family by performing a focused short-read NGS data reanalysis and identifying “dark” genomic regions overlapping chrX genes highly associated with clinical features shared between affected family members. The ARXdup24 variant, validated in all patients tested so far, supports genotype–phenotype correlation in the first Partington syndrome family in Cyprus. This study highlights that examining underrepresented genomic regions and using phenotype-driven tools can provide critical diagnostic insights in unresolved XLID cases. We anticipate that a similar focused reanalysis approach can be successfully applied in additional unresolved cases of XLID or other conditions, to end the long and challenging diagnostic journey of patients, without the need for additional experimental procedures, leading to accurate diagnosis, management and counselling. These studies will collectively shed more light and potentially unravel the role of the “dark” genome in human diseases.

## Figures and Tables

**Table 1:** Clinical description and initial diagnostic testing results of the affected siblings III:3 and III:4.

Phenotype/ Initial Test	Case III:3	Case III:4
Abnormality of mental function	Mild-moderate intellectual disability, psychiatric abnormalities, irritability and anger tendencies.	Mild-moderate intellectual disability.
Abnormal brain morphology	Brain atrophy (signs of bilateral hippocampal atrophy) evidenced at the age of 56.	NA ^1^
Seizures	Yes, generalised tonic–clonic (grand mal) seizures initiating at the age of 3. Last reported seizure at the age of 22. Seizures were controlled with antiepileptic drugs.	No
Abnormality of movement	Focal (hand) dystonia and gait disturbance.	Focal (hand) dystonia and hyperreflexia.
Abnormal speech pattern	Dysarthria	NA ^1^
Dysmorphic features	Relatively large ears, wide nasal base with broad nasal tip.	Protruding large ears, pinched nose.
Chromosomal analysis	Apparently normal, 46,XY	Apparently normal, 46,XY
chrX-specific array/ Array-CGH	Apparently normal constitution	Apparently normal constitution
Fragile X testing	Negative (30 CGG repeats)	Negative (21 CGG repeats)
WES/WGS	Negative	Negative

^1^ NA = information not available.

## Data Availability

The original contributions presented in this study are included in the article/supplementary material. Further inquiries can be directed to the corresponding author. The reported pathogenic *ARX* duplication NM_139058.3:c.441_464dup p.(Ala148_Ala155dup) was submitted to the freely accessible, public database of human genetic variants ClinVar (www.ncbi.nlm.nih.gov/clinvar/) (ClinVar accession number: SCV006327456) [58].

## Supplementary Materials

The following supporting information can be downloaded at: https://www.mdpi.com/article/10.3390/genes16101224/s1, File S1: Dark x Phenomizer.

## Abbreviations

The following abbreviations are used in this manuscript:

ALG13	ALG13 UDP-N-acetylglucosaminyltransferase subunit
array-CGH	Array-Comparative Genomic Hybridization
ARX	Aristaless-related homeobox
CDS	Coding DNA sequences
chr	chromosome
CNV	Copy number variant
FISH	Fluorescence in situ hybridization
FMR1	Fragile X Messenger Ribonucleoprotein 1
FXS	Fragile X syndrome
HGVS	Human genome variation society
HPO	Human phenotype ontology
ID	Intellectual disability
IGV	Integrative genomics viewer
indel	Insertion/deletion
KDM5C	Lysine-specific demethylase 5C
NGS	Next-generation sequencing
SMN1	Survival of motor neuron 1
SMN2	Survival of motor neuron 2
SNV	Single-nucleotide variant
TBX1	T-box transcription factor 1
WES	Whole-exome sequencing
WGS	Whole-genome sequencing
XLID	X-linked intellectual disability
